# A rare case of intervertebral disc calcification combined with ossification of the posterior longitudinal ligament in a child: a case report and literature review

**DOI:** 10.1186/s12891-024-07218-2

**Published:** 2024-02-09

**Authors:** Cheng Ye, Mingliang Shi, Dong Xie, Hao Wu, Qing Chen, Lili Yang

**Affiliations:** 1 Department of Orthopaedics, Spine Center, Shanghai Changzheng Hospital, Second Affiliated Hospital of Naval Medical University, Shanghai, 200003 China; 2 Department of Orthopaedics, No. 905 Hospital of PLA Navy, Shanghai, China

**Keywords:** Intervertebral disc calcification, Ossification of the posterior longitudinal ligament, Diagnosis, Treatment, Case report

## Abstract

**Background:**

Intervertebral disc calcification (IDC) combined with calcification in children has been sporadically reported, while ossification of the posterior longitudinal ligament (OPLL) in the cervical spine in pediatric patients is exceedingly rare. The aim of this study is to investigate the potential prognosis and outcomes associated with this condition.

**Case presentation:**

We present an unusual case involving a 10-year-old Chinese child diagnosed with calcified cervical disc herniation and ossification of the posterior longitudinal ligament. Conservative treatment measures were implemented, and at the 1-month and 6-month follow-up, the patient's pain exhibited significant improvement. Subsequent cervical MRI and CT scans revealed the complete disappearance of OPLL and substantial absorption of the calcified disc. During the three-month follow-up, CT demonstrated slight residual disc calcification, however, the patient remained asymptomatic with no discernible limitation in cervical motion.

**Conclusions:**

We conducted a comprehensive review of several cases presenting with the same diagnosis. It is noteworthy that IDC combined with OPLL in children constitutes a rare clinical entity. Despite imaging indications of potential spinal canal occupation, the majority of such cases demonstrate complete absorption following conservative treatment, with OPLL exhibiting a faster absorption rate than calcified discs.

## Background

Intervertebral disc calcification (IDC) in children is a rare phenomenon. Since the initial report by Baron in 1942, more than 300 cases have been documented [1]. It is characterized by calcification of the nucleus pulposus at one or more levels of the intervertebral disc, with the lower cervical spine commonly affected. Etiologically, IDC can be attributed to genetics factors, infection-related inflammatory reactions, trauma, or nutritional metabolic deficiencies. Nevertheless, the majority of cases exhibit a self-limiting nature, offering a favorable prognosis, thus obviating the necessity for invasive diagnostic procedures, such as biopsy [2]. In contrast, ossification of the posterior longitudinal ligament (OPLL) is commonly seen in adults. Its pathological mechanism involves heterotopic ossification of the posterior longitudinal ligament, which may result in compression of the spinal cord and nerve roots, thereby giving rise to various clinical symptoms and signs. The onset of OPLL is insidious and often goes unnoticed by patients until they experience a sudden decline in muscle strength or even paralysis.

IDC combined with OPLL in children is exceedingly uncommon, with only seven reported cases [3–9]. While conservative treatment has been predominantly employed in the majority of these cases, a standardized treatment protocol for this condition is currently lacking. Through the presentation of a new clinical case and a comprehensive review of pertinent literature, our objective is to augment physicians' diagnostic and treatment proficiency. Simultaneously, we aim to deepen our comprehension of the potential pathological mechanisms, treatment options, and the prospects for regression and prognosis associated with this uncommon condition..

## Case presentation

This 10-year-old boy suffered from neck and shoulder pain along with restricted movement including difficulty bowing and rotating his head, persisting for one month. He specifically noted heightened pain on the left side, exacerbated during prolonged periods of reading, registering a visual analogue scale (VAS) score of 4. The patient denied neck trauma, fever, and infection, oral medication use, and any history of other diseases. There was no identified familial history of OPLL in the relevant family history. Upon physical examination, the patient’s neck exhibited limited flexion and lateral rotation, while maintaining normal muscle strength in the extremities with no discernible neurological abnormalities. Laboratory findings revealed an eosinophil count of 0.6 × 10^9^/L, slightly exceeding than the normal range (0–0.5 × 10^9^/L). Other parameters such as blood calcium level (2.35 mmol/L), erythrocyte sedimentation rate (ESR), C-reactive protein (CRP), and rheumatoid immune-related indexes was within the normal range.

**Fig. 1:**
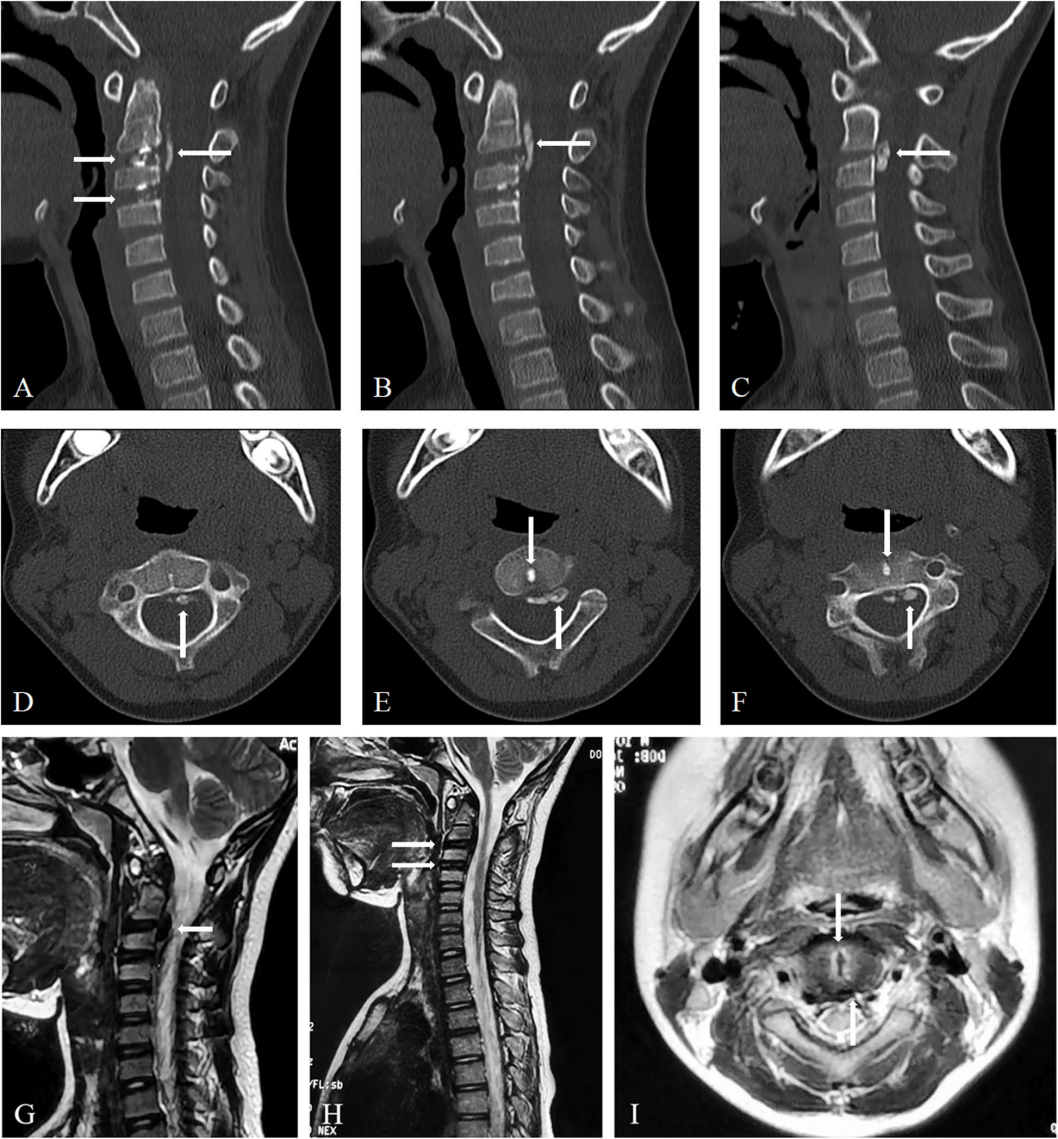
**A**-**F** CT images of cervical vertebra depict intervertebral disc calcification at the C2/3 and C3/4 levels, with protrusion of OPLL at posterior edge of the C2 vertebral body and the C2/3 intervertebral disc level, encroaching into the spinal canal; **G**-**I** MRI scans of cervical spine demonstrate a reduction in signal intensity in T2WI for the C2/3 and C3/4 intervertebral discs, as well as the posterior longitudinal ligament of the C2 vertebral body. In the transverse plane, the PLL at the C2/3 level exhibits central-left herniation, resulting in slight compression of the dural sac

**Fig. 2:**
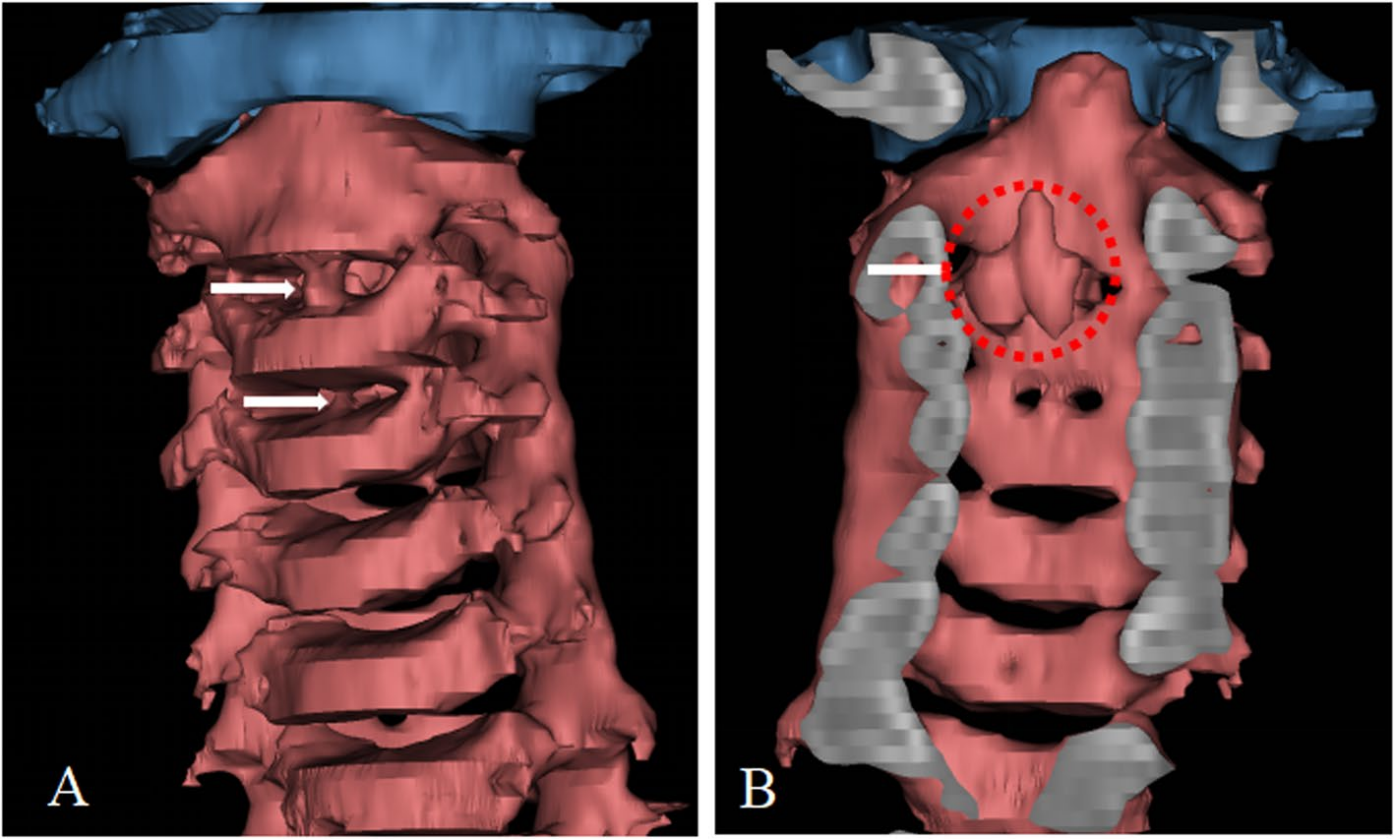
**A** Intervertebral disc calcification is observed at C2/3 and C3/4 levels; **B** Sectional view shows finger-like protrusion of OPLL extending from the posterior edge of C2 vertebral body and C2/3 intervertebral disc level into spinal canal

Three-dimensional CT reconstruction indicated calcification of the intervertebral disc at the C2/3 and C3/4 levels, along with OPLL situated at the lateral edge of the C2 vertebral body and the C2/3 disc level. MRI in the sagittal position on T2WI revealed diminished signal intensity in the posterior longitudinal ligaments of the C2/3 and C3/4 discs, as well as the posterior border of the C2 vertebral body. In the transverse plane, the posterior longitudinal ligament at the C2/3 level exhibited central-left herniation, resulting in compression of the dural sac (Fig. 1). The spinal canal occupancy measured by using the imag J was approximately 30.30%. Furthermore, Materialise Mimics software was employed to convert the OPLL image of this patient into a three-dimensional model (Fig. 2), which clearly illustrated the morphology, precise location, and dimensions of both the OPLL and the calcified disc.

**Fig. 3:**
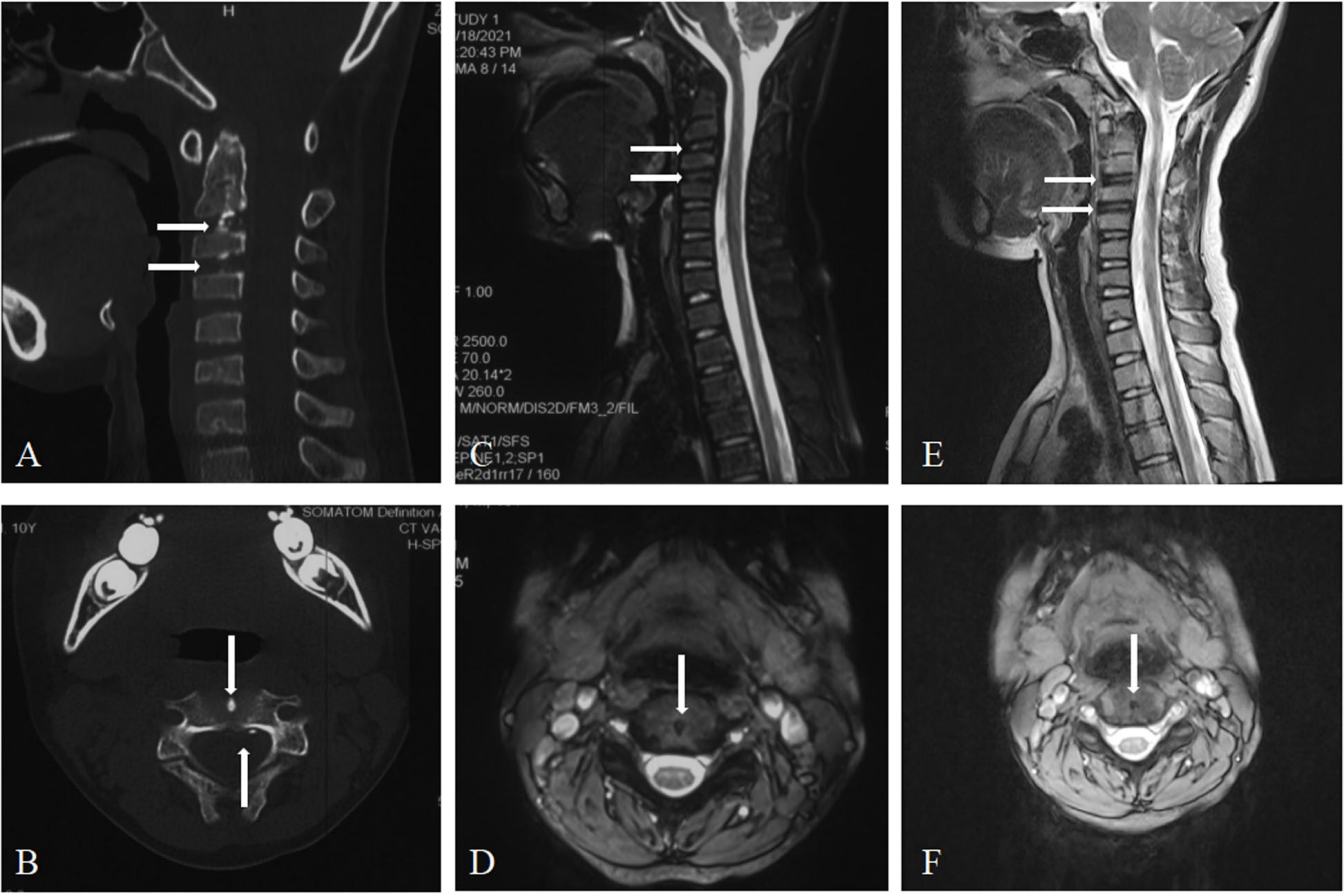
Radiological imaging at one month. **A**, **B **CT images reveal the absorption of IDC at the C2/3 and C3/4 levels after 1-month follow-up, and the OPLL at the posterior edge of C2 vertebral body and C2/3 disc level completely disappeared; **C**, **D **After 1-month follow-up, The transverse view showed alleviation of the compression on the dural sac at the C2/3 level,with residual calcification foci still present at the intervertebral disc level; **E**, **F **At the 6-month follow-up, MRI reveals increased signals in the C2/3 and C3/4 disc compared to the initial scans, indicative of reduced calcification

The diagnosis of IDC with OPLL was confirmed based on the patient's medical history and imaging examination. Considering the absence of current symptoms indicative of spinal nerve function impairment and the patient's tolerable pain level, conservative treatment measures were adopted. These measures included cervical brace protection and the administration of small doses of oral NSAIDs. The child experienced significant relief from neck and shoulder pain after one-month follow-up. Subsequent cervical MRI and CT scans revealed that the complete disappearance of OPLL and the calcified disc herniation showed substantial absorption. During the six-month follow-up, CT imaging indicated slight residual disc calcification, yet the patient remained asymptomatic without any any limitation of cervical motion (Fig. 3).

## Discussion and conclusions

**Fig. 4:**
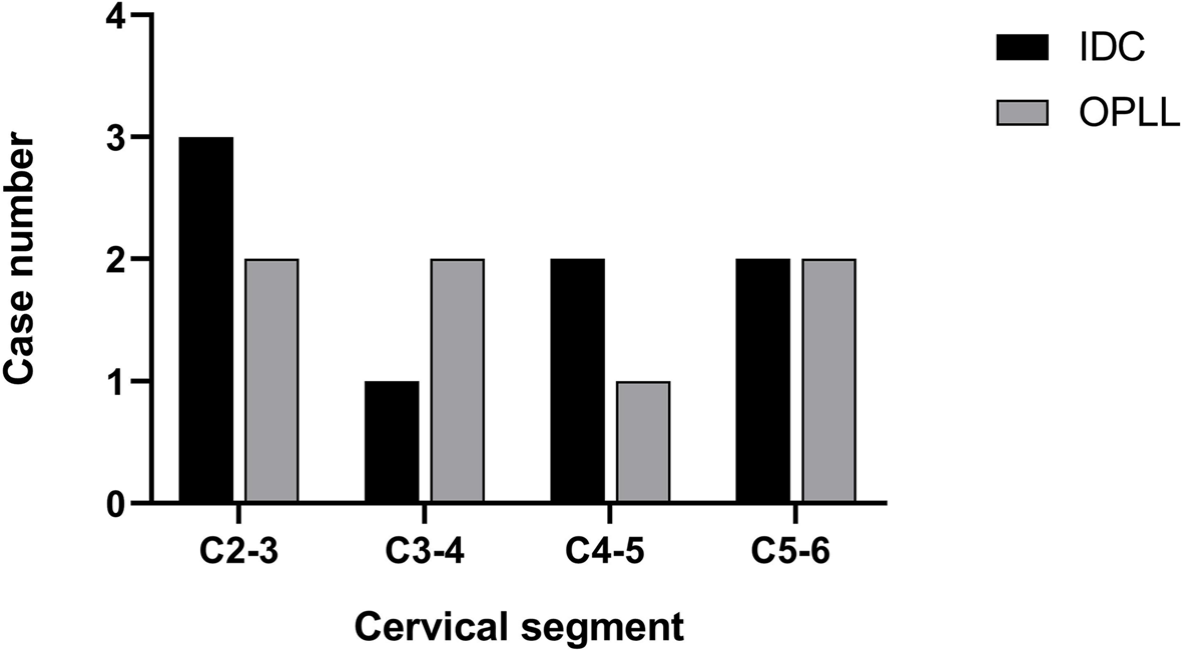
Distribution of IDC and OPLL levels in literature reports

We conducted a comprehensive review of the existing literature to cervical IDC combined with OPLL in pediatric cases identifying a total of seven reported instances (Table 1). Our analysis revealed a male-to-female ratio of 3:4, with an age range spanning 6 to 11 years. The ethnic distribution demonstrated six cases among Asians and one among American. The prevailing clinical manifestations included neck and shoulder pain along with limited cervical movement, while one case presented with spinal radicular symptoms. Only two patients had a history of cervical spine trauma, and none exhibited recent infections. No noteworthy alterations were observed in the recorded laboratory indices. Upon evaluation of imaging data, it was observed that the majority of the disc calcification were single interstitial, predominantly located at the C2-C3 level (*n* = 3, 37.5%), C3-C4 level (*n* = 1, 12.5%), C4-C5 level (*n* = 2, 25.0%), and C5-C6 level (*n* = 2, 25.0%). The distribution of OPLL was identified as primarily singular, manifesting at the C2-C3 level (*n* = 2, 28.57%), C3-C4 level (*n* = 2, 28.57%), C4-C5 level (*n* = 1, 14.29%), and C5-C6 level (*n* = 2, 28.57%) (Fig. 4). In our presented case, the calcified disc was identified across two segments, whereas the OPLL exhibited a continuous pattern along the posterior border of the C2 vertebral body and, less commonly, a nodular pattern at the C2/3 level.

**Table 1:** Data of the reported cases of IDC with OPLL in literature

Case	Time	Sex	Nationality	Age	Symptoms	Trauma	Laboratory index	IDC level	OPLL level	Spinal canal occupancy	Povlve ratio	Treatment Plan	Follow-up time	Prognosis
Zhu J:Sun K:Xu X.etc	2019	F	CHINA	8	Neck and back pain, radiating pain, and numbness in the left upper limb	No	Normal	C5/6	C5/6	39.56%	0.79	Non-intervention	6 m	Complete absorption of OPLL and IDC
Wang G;Kang Y;Chen F.etc	2016	F	CHINA	11	Neck pain for 3 months, aggravated for half a month, numbness in the left arm	No	Normal	C5/6	C5/6	47.15%	0.91	Bed rest, NSAIDs, intermittent cervical traction, cervical gear protection	6 m	IDC and OPLL significantly reduced after two months
Du JJ; Chen YF; Peng Y.etc	2018	M	CHINA	6	Neck pain with a VAS score of 7	No	ESR increased (69 mm/h) CRP increased (11.80 mg/L)	C2/3	C3/4	39.37%	0.97	2 weeks of analgesic treatment, intermittent cervical traction	9y	OPLL disappear and IDC remains
Wang XD;Su XJ;Chen YK.etc	2021	M	CHINA	6	Serious neck pain and stiffness	Neck injury while dancing 3 days ago	Normal	C2/3	C2/3	60.42%	0.83	cervical gear protection	3 m	Significant recovery within 3 weeks, 0PLL disappears and IDC remains
Mizukawa K:Kobayashi T;Yamada N	2017	F	JAPAN	6	Neck pain, limited range of motion, pain increases with stretching	No	WBC normal(8600/L) CRP slightly increased (1.5 mg/dL)	C4/5	C4/5	40.82%	1.06	1 week of rest and oral acetyl chlorophenol	6 m	OPLL and IDC disappeared after 6 months on x-ray
Li CH:Lui TH;Ngai WK;	2016	F	CHINA	7	Pain around the left posterior paravertebral muscle worsens with activity	No	Mildly elevated white blood cell count and inflammatory markers	C3/4、C4/5	C3/4	22.13%	0.98	panadol antipyretic and cervical gear protection	1y	OPLL disappeared and IDC decreased after 2 months
Dell MC; Flores M; Murray JV Jr;	2016	M	USA	9	Neck pain and stiffness for 3 months	Neck pain got worse after an injury during soccer 3 days ago	Thalassemia	C2/3	C2/3	44.83%	0.97	Anti-inflammatory drug	3 m	Basic disappearance of OPLL with residual IDC

This table depicts the features of the published cases of cervical IDC combined with OPLL in children

Since the first case of intervertebral IDC was reported by Baron in 1942, approximately 300 cases have been documented in the literature [1]. IDC is characterized by the calcification of the nucleus pulposus at one or more levels, often occurring in the lower cervical spine. Many patients are incidentally discovered through routine X-rays, while others present with nonspecific symptoms, including neck pain, limited cervical motion, and torticollis deformity [10]. Predominantly, neck and shoulder pain manifest as the most common clinical symptom in affected individuals [11].

IDC combined with OPLL in children is a rare condition with a complex pathophysiology that involves multifactorial processes. While the precise etiological basis remains incompletely understood, several contributing factors have been proposed, such as infection, trauma, and related pathological influences have been proposed as potential contributors to its development. Inflammatory mediators, such as histamine, tumor necrosis factor-α (TNF-α), interleukins (ILs), and vascular endothelial growth factor (VEGF), are thought to play a role. The inflammatory response triggered by calcified herniated discs may induce an inflammatory milieu in the posterior longitudinal ligament, promoting endochondral osteogenesis. Mechanical stress resulting from disc calcification may cause stress changes in adjacent segments, contributing to the development of OPLL. This is particularly relevant in the cervical spine, where mechanical stresses are significant. Notably, the erythrocyte sedimentation rate (ESR) stands out as the most sensitive indicator of IDC progression [11]. Schmit Pet al. reported two specific cases involving IDC patients, further shedding light on potential contributing factors. One case involved a child with a history of early corticosteroid treatment for rheumatoid arthritis, while the other child with Williams and Van Beuren syndrome. These instances underscore the plausible role of related pathological factors in the genesis of IDC [12].

OPLL refers to the abnormal ectopic ossification occurring in the posterior longitudinal ligament of the cervical spine. This condition can result in a restricted range of motion in the cervical spine and compression of the spinal cord [13]. Common symptoms of OPLL patients encompass neck pain, dysfunction in limb movement or sensation, and sphincter dysfunction. Surgical intervention may become necessary in severe cases. The pathological mechanism underlying OPLL entails the ectopic ossification of the posterior longitudinal ligament, a process influenced by both genetic factors and non-genetic factors. Non-genetic contributors include dietary factors, obesity, mechanical stress stimulation, trauma-induced ligament injury, and hormone levels. Distinguishing between ossification and calcification of the PLL often involves a combination of clinical evaluation, imaging studies, and sometimes laboratory tests. Typically OPLL appears as continuous or segmental ossification along the posterior longitudinal ligament, creating a bony mass, and usually reveals a continuous low-signal intensity mass on T1-weighted images, corresponding to the ossified ligament. Differently, the calcification may show discrete calcified deposits within the ligament, often without forming a continuous bony mass, and typically manifests as areas of signal void or low-signal intensity within the ligament on various sequences, representing calcified deposits. In some cases, laboratory tests measuring specific biochemical markers related to bone metabolism may provide additional information. However, this is less commonly used and typically reserved for cases where there is uncertainty or suspicion of an underlying metabolic disorder. In rare cases where a definitive diagnosis is challenging, a biopsy of the affected ligament may be considered. However, due to the invasive nature of this procedure and the risk involved, it is usually not the first choice.

Cai [14] and Kawaguchi [15] conducted a comparative analysis of plasma biomarker in patients with OPLL and healthy volunteers, differentiating between those with progressive and non-progressive OPLL. In OPLL patients, elevated levels of serum fibroblast growth factor-23 (FGF-23) and high-sensitivity C-reactive protein (hs-CRP) were observed, accompanied by a significant reduction in serum phosphorus values. Moreover, concentrations of FGF-23 and hs-CRP in the ossification progression group exceeded those in the progression-free group. These findings suggests the involvement of phosphorus metabolism, inflammatory response, and FGF-23 in the initiation and progression of OPLL. Matsunaga [16] reported that the area most commonly affected by the progression of OPLL is the region characterized by disc stretching and distortion. Zhang [17] conducted isolated cells extracted from OPLL patients demonstrated that mechanical stress markedly decreased vimentin expression while increasing the expression of osteocalcin (OCN), alkaline phosphatase (ALP), and COL I in the ligament cells of OPLL patients. This in turn facilitated the osteogenic differentiation and triggered OPLL development. The prevalence of OPLL varies geographically, with rates of 1.8%-4.1% in Japan, 0.2%-1.8% in China, and only 0.01%-1.7% in the United States and Europe [18]. The male-to-female ratio is approximately 2:1 [19].

The anatomical composition of the intervertebral disc is marked by the peripheral annulus fibrous and the central nucleus pulposus. Notably, blood vessels are typically absent, except for the outer third of the annulus fibrosus, which contains a low number of cells [20]. In instances of intervertebral disc degeneration, stimulation induced by inflammatory mediators, such as histamine, tumor necrosis factor α (TNF-α), interleukins (ILs), vascular endothelial growth factor (VEGF), and neovascularization occurs within the disc [21]. This stimulation leads to the differentiation of monocytes into macrophages, triggering an increase in the release of matrix metalloproteinases (MMPs). This heightened release facilitates the process of disc calcification phagocytosis and resorption [22]. In contrast to the inability to resorb the abnormal ossification of adult OPLL, the anatomical characteristics of the intervertebral disc and the process of degenerative reabsorption contribute to a slower resorption of calcification.

In our ongoing patient follow-up investigation, coupled with a review of the prognoses of the remaining seven children with IDC combined with OPLL reported in the literature, a noteworthy observation emerged. It was consistently observed across all cases that the resorption of OPLL occurred earlier than the resorption of disc calcification. Specifically, in the current case, complete disappearance of OPLL was observed after merely 1 month. Furthermore, after 6 months mark, substantial resorption of the patient’s IDC was noted. In contrast, a case reported by Du [7] and others, where the patient with lingering disc calcification even after 9 years of follow-up. This discrepancy underscores the significantly slower rate of IDC resorption compared to that of OPLL.

In consideration of the absence of reported cases involving irreversible spinal cord function damage solely due to OPLL in children, we propose a hypothesis that OPLL in children may be secondary to a specific state of IDC. This speculation is rooted in the concept that an inflammatory response within the posterior longitudinal ligament, thereby instigating the accumulation of inflammatory or immune factors. These factors, in turn, induce endochondral osteogenesis in the posterior longitudinal ligament [11]. The inflammation levels including CRP and ESR observed in relevant case reports may potentially reflect this inflammatory response process [5–7]. Simultaneously, the calcification of intervertebral discs induces stress changes in the corresponding disc segments,, leading to an unstable state in the spine. This state of spinal instability accelerates the progression of OPLL. Unlike adult OPLL, the OPLL observed in children is still in an evolving state, characterized by an immature level of osteogenesis that is susceptible to resorption. This susceptibility is attributed to the high metabolic levels in children and the upregulation of blood supply resulting from inflammation around the ossification. These findings provide a promising avenue for further research in this domain.

Neck and shoulder pain, stiffness, and limited motion represent the primary symptoms observed in the initial stages of IDC combined with OPLL in most pediatric cases, with severe neurological impairment of the spinal cord being a rare occurrence. In our study, we conducted a comprehensive review of OPLL morphology and spinal canal encroachment in various cases. However, the actual parameters such as OPLL thickness haven’t been measured in some cases due to the absence of a relevant scale in the provided imaging data. To address this limitation, we employed the imag J software for precise measurements, focusing on the Pavlov ratio and spinal canal occupancy. Our results revealed a range of spinal canal occupation rates from 22.13–60.42%, with an average of 42.04 ± 11.42%. Additionally, the Pavlov ratios ranged from 0.79 to 1.06, with a mean of 0.93 ± 0.09. Interestingly, despite significant OPLL invasion, most children did not exhibit severe neurological impairment of the spinal cord. This phenomenon may be attributed to the larger effective space within the spinal canal in children, as well as to the fact the early stage of ossification of children's OPLL has a softer texture compared to adults'. Furthermore, unlike in adults, our examination of imaging characteristics highlighted a distinct pattern that the majority of OPLL in children was predominantly situated in the segment where IDC occurred, and the morphology of the OPLL protruding into the spinal canal exhibited rounded and smooth appearance, devoid of osteophytes or bone bridges evident in the vertebral body of this segment. These findings strongly suggest that children's OPLL is more likely to develop secondary to IDC. Furthermore, the smooth morphology of the OPLL avoids impairing spinal cord function, resulting in milder clinical symptoms, a notable contrast to the rapid decline in spinal cord function typically observed in adults with OPLL.

When children present with complains of neck and shoulder pain, limited mobility, or radicular symptoms lacking an obvious cause, the possibility of IDC children combined with OPLL should be considered. The distribution of this disease appears to demonstrate age-specific and race-specific skew. CT is the preferred diagnostic modality for IDC with OPLL, but it is not the primary choice for long-term follow-up in children due to the radioactivity [23]. Digital radiography (DR) and MRI can be used as examination methods to assess the extent of IDC and OPLL and spinal cord compression. Presently, conservative treatment stands as the preferred approach for IDC combined with OPLL, particularly in cases where there are no signs of spinal nerve impairment and the patient's pain is tolerable. The rationale for conservative management lies in the generally positive outcomes observed, including the rapid reabsorption of OPLL. It aims to alleviate symptoms, prevent neurodeficits, and avoid the potential risks associated with surgical interventions. Conservative treatment measures such as cervical brace protection and oral non-steroidal drugs, can be employed with generally favorable outcomes. Zhu et al. [8] suggested that the outcome of the disease is generally positive by studying patients with non-interventional treatment. In our cohort, no significant narrowing of the vertebral space, cervical instability, or hypertrophic folds of the ligamentum flavum were observed, the rarity of significant neurodeficits in the literature supports the efficacy of conservative management. Due to uncertain effects and the potential risk of exacerbating spinal cord injury in the traction state with heavy intraspinal compression [24], we do not recommend traction treatment for patients. Surgical intervention is considered cautiously and reserved for cases with a rapid decline in spinal cord nerve function. The decision for surgery depends on the severity of neurological impairment, the presence of compression, and individual patient factors. Potential surgical procedures include decompression and stabilization to address spinal cord compression [25]. However, surgery is associated with inherent risks, and the decision to proceed should be carefully weighed against the potential benefits.

While OPLL may extensively encroach upon the spinal canal, the resultant impairment of spinal cord function is generally mild, and after conservative treatment, reabsorption of OPLL tends to be rapid, leading to a positive prognosis. It is noteworthy that residual intervertebral disc calcification may persist, prompting the need for further investigation into its potential role in accelerating disc degeneration and contributing to the recurrence of OPLL in adults. In this presented case, we treated the child with cervical brace protection and a limited course of oral NSAIDs, effectively alleviating his pain symptoms but also mitigated the risk of unforeseen factors causing a rapid deterioration of spinal cord function. The overall prognosis for the patient was deemed quite favorable. In our perspective, surgical intervention should approached cautiously and reserved for cases where patients experience a rapid decline in spinal cord nerve function, warranting careful consideration of the potential benefits and risks associated with such interventions.

## Data Availability

This is a case report of a single patient, to protect privacy and respect confidentiality; none of the raw data has been made available in any public repository. The original reports, laboratory studies, imaging studies, and outpatient clinic records are retained as per normal procedure within the medical records of our institution.The datasets and materials supporting the conclusions of this article are included within the article, and further inquiries can be directed to the corresponding author.

## Abbreviations

IDC: Intervertebral Disc Calcification
OPLL: Ossification of the Posterior Longitudinal Ligament
ESR: Erythrocyte sedimentation rate
FGF-23: Factor-23
hs-CRP: High-sensitivity C-reactive protein
CT: Computer tomography
MRI: Magnetic resonance imaging
DR: Digital radiography
MMPs: Matrix metall oproteinases
TNF-α: Tumor necrosis factor α
ILs: Interleukins
VEGF: Vascular endothelial growth factor
OCN: Osteocalcin
ALP: Alkaline phosphatase
